# Residential proximity to nuclear power plants and cancer incidence in Massachusetts, USA (2000–2018)

**DOI:** 10.1186/s12940-025-01248-6

**Published:** 2025-12-18

**Authors:** Yazan Alwadi, John S. Evans, Joel Schwartz, Carolina L. Zilli Vieira, David C. Christiani, Brent A. Coull, Petros Koutrakis

**Affiliations:** 1 Department of Environmental Health, Harvard T.H. Chan School of Public Health, 677 Huntington Ave, Boston, MA 02115 USA; 2 Division of Pulmonary and Critical Care Medicine, Department of Medicine, Massachusetts General Hospital and Harvard Medical School, Boston, MA. 02114 USA; 3 Department of Biostatistics, Harvard T.H. Chan School of Public Health, Boston, MA USA

**Keywords:** Nuclear power plants, Radioactive emissions, Cancer incidence, Relative risk, Proximity analysis, Massachusetts, Environmental epidemiology

## Abstract

**Purpose:**

To investigate the associations between residential proximity to nuclear power plants and ZIP code–level cancer incidence among Massachusetts residents.

**Methods:**

We assessed proximity of Massachusetts ZIP codes to nuclear power plants using an inverse-distance weighted metric. We obtained cancer incidence data (2000–2018) from the Massachusetts Cancer Registry. We applied two approaches: (1) longitudinal Generalized Estimating Equation (GEE) Poisson regression to evaluate yearly incidences for all cancers combined, and (2) cross-sectional log-linear Poisson regression for site-specific cancers. We adjusted models for PM2.5, demographic, socioeconomic, environmental, and healthcare covariates, and stratified analyses by sex and four age groups (45–54, 55–64, 65–74, 75 +).

**Results:**

Proximity to plants significantly increased cancer incidence, with risk declining by distance. At 2 km, females showed RRs of 1.52 (95% CI: 1.20–1.94) for ages 55–64, 2.00 (1.59–2.52) for 65–74, and 2.53 (1.98–3.22) for 75 + . Males showed RRs of 1.97 (1.57–2.48), 1.75 (1.42–2.16), and 1.63 (1.29–2.06), respectively. Cancer site-specific analyses showed significant associations for lung, prostate, breast, colorectal, bladder, melanoma, leukemia, thyroid, uterine, kidney, laryngeal, pancreatic, oral, esophageal, and Hodgkin lymphoma, with variation by sex and age. We estimated 10,815 female and 9,803 male cancer cases attributable to proximity, corresponding to attributable fractions of 4.1% (95% CI: 2.4–5.7%) and 3.5% (95% CI: 1.8–5.2%).

**Conclusions:**

Residential proximity to nuclear plants in Massachusetts is associated with elevated cancer risks, particularly among older adults, underscoring the need for continued epidemiologic monitoring amid renewed interest in nuclear energy.

**Supplementary Information:**

The online version contains supplementary material available at 10.1186/s12940-025-01248-6.

## Introduction

Nuclear energy has been a cornerstone of the U.S. electricity landscape since the launch of its first commercial nuclear power plant in 1958. As of August 2023, the United States remains the world’s largest producer of nuclear electricity, with 93 operational reactors across 54 plants in 28 states. Despite a significant reduction in the number of reactors from a peak of 112 in 1990 to 93 in 2022 [25], nuclear power consistently contributes around 18–20% of the nation’s total electricity generation and over 50% of its carbon-free electricity (WNA, U.S. Energy Information Administration (EIA)).

In 2023, the startup of Vogtle Unit 3 marked the first new U.S. nuclear reactor to begin commercial operation in over three decades, reflecting renewed interest in nuclear energy. Amid growing concerns over energy security and the urgent need to decarbonize the power sector, interest in nuclear power has increased, particularly in advanced reactor technologies and small modular reactors (SMRs). Federal initiatives such as the Inflation Reduction Act of 2022 and the 2025 executive order to quadruple U.S. nuclear capacity by 2050 have further fueled this momentum. While significant challenges remain, these policy signals suggest that nuclear energy is expected to play an expanding role in the future U.S. electricity mix (WNA; U.S. Energy Information Administration).

However, while this anticipated expansion underscores nuclear power’s potential contributions to achieving decarbonization goals, the health and environmental implications of increased reliance on nuclear technology warrant careful consideration, particularly due to radioactive pollutants emitted from nuclear power plants. These pollutants can contaminate water, air, soil, and crops, exposing populations through inhalation, ingestion, and dermal contact with primary emissions. Exposure can persist over time, for example, through radiation emitted from contaminated soil (also known as ground-shine emissions) [12]. Human exposure pathways depend largely on the transport medium-for instance, air can carry these pollutants as radioactive particulate matter and gases. Radiation from the decay of radionuclides emitted by nuclear plants has been extensively studied and is a well-established risk factor for multiple cancers [4, 9, 13, 14, 17, 27].

Given these potential health implications, understanding the epidemiologic evidence related to nuclear power plant emissions is crucial to inform policy and public health interventions. Despite the widespread reliance on nuclear power in the U.S., epidemiologic research investigating the health impacts of nuclear power plants remains relatively limited, and existing studies worldwide have produced heterogeneous results. Some studies conducted internationally have reported that proximity to nuclear power plants has no impact on cancer risk [1, 6–8, 11, 18, 20, 21], whereas others have identified significant associations [5, 15, 23, 26, 28, 29].

Given the mixed international epidemiologic evidence and the resurging interest in expanding nuclear power in the U.S., detailed analyses are increasingly crucial for accurately assessing the potential health risks associated with proximity to nuclear power plants. Previous ecological, national-level analyses conducted by our group (currently under review), which used the distance from the geographic center of each county to the nearest nuclear power plant, found consistently positive associations with all-cancer mortality, as well as lung, breast, and colorectal cancer mortalities across the United States [2, 3]. However, these national studies were conducted using data aggregated at the county level and relied on cancer mortality rather than incidence data, which may underestimate the full burden of cancer related to nuclear plant emissions.

Massachusetts (MA) represents an ideal setting for addressing these limitations as its residents live within approximately 120 km of seven nuclear power plants: Pilgrim Nuclear Power Station, Seabrook Station, Vermont Yankee, Millstone Power Station, Indian Point Energy Center, Connecticut Yankee, and Yankee Rowe. Additionally, Massachusetts maintains one of the longest and most comprehensive population-based cancer registries in the United States (the Massachusetts Cancer Registry, MCR), which systematically collects detailed cancer incidence data. The availability of this high-quality resource enhances the ability to rigorously evaluate associations between nuclear plant emissions and cancer risks at finer spatial levels.

In this study, we use Massachusetts’ cancer registry data at the ZIP code level to examine the association between residential proximity to nuclear power plants and cancer incidence. Building on our prior national mortality analyses at the county level, this study aims to provide additional evidence to support the evaluation of a potential causal relationship by examining the association using a different health outcome and finer spatial resolution. The findings aim to inform regulatory policy and guide future research on the potential health effects of nuclear power plants emissions amid renewed national interest in nuclear energy.

## Data

### Cancer incidence

Cancer incidence data by ZIP Code for the years 2000 to 2018 were obtained from the Massachusetts Cancer Registry (MCR), managed by the Massachusetts Department of Public Health's Office of Population Health (2024). The MCR collects reports of newly diagnosed cancer cases from healthcare facilities and practitioners across the state. Each year, the North American Association of Central Cancer Registries (NAACCR) reviews cancer registry data for quality, completeness, and timeliness. For 2010–2014, the MCR’s annual case count was estimated by NAACCR to be more than 95% complete for each year. The MCR has achieved the gold standard for this certification element as well as for six other certification elements for each case year since 1997. The data set included the following cancers: oral, esophageal, stomach, colorectal, pancreas, larynx, lung, melanoma, breast, cervix, uterine, prostate, testes, bladder, kidney, brain/NS-invasive, thyroid, Hodgkin lymphoma (HL), myeloma, and leukemia.

Tables 1S and 4S in the Supplementary Materials present the total and mean annual number of cancer cases, stratified by cancer type, sex, and age group, providing a detailed epidemiologic overview of the study population.

### Covariates

We adjusted for a set of annual ZIP code–level covariates (2000–2018) that may confound the association between proximity to nuclear power plants and cancer incidence or may be independently associated with cancer risk. None of these covariates are hypothesized to lie on the causal pathway between nuclear power plants proximity and outcome and are therefore not considered mediators. The covariates include educational attainment, PM2.5 [30], median household income, poverty rate, racial composition (White, Asian, African American), population density, yearly average temperature & relative humidity, smoking prevalence, proximity to the nearest hospital, percentage of population over age 65, age distribution, and rental housing proportion. Full definitions and sources are provided in Supplemental Table 3S. We calculated the age group and sex specific populations for each ZIP code using population grided data sourced from Worldpop [31].

### Nuclear power plants

We obtained data on nuclear power plant locations and operational timelines from the U.S. Energy Information Administration (EIA). For each ZIP code population center in Massachusetts, we identified all nuclear power plants located within a 120-km radius. A total of seven nuclear facilities met this criterion: Connecticut Yankee (CT), Millstone (CT), Vermont Yankee (VT), Yankee Rowe (MA), Seabrook Station (NH), Indian Point (NY), and Pilgrim (MA). The operational periods for each facility are detailed in Supplemental Table 4S.

## Methods

### Nuclear power plants proximity assessment

We calculated the annual proximity of each ZIP code to nuclear power plants by summing the inverse distances (1/d, in kilometers) from all facilities that were both operational in a given year and located within 120 km of the ZIP code’s population center. For years in which a plant was not operational, its 1/d contribution was set to zero. This formulation inherently accounts for overlapping exposure zones, as ZIP codes located between multiple plants receive higher cumulative proximity values reflecting combined influence from more than one facility. This continuous, distance-weighted metric provides a direct measure of cumulative proximity, with higher values reflecting closer and/or more numerous nearby nuclear facilities. The operational timelines of all included plants are provided in Table 4S of the Supplemental Materials.

### GEE longitudinal model of all cancers

We calculated the sum of all cancer cases for each ZIP code, year, sex, and age group (45–54, 55–64, 65–74, and 75 + years) because these age groups had sufficient case counts to produce stable rates within a spatiotemporal analytical framework. Supplemental Table 2S presents the total number of cancer cases, as well as the mean annual number of cases, stratified by sex and age group.

We modeled the number of all cancer cases from 2000 to 2018 across all ZIP codes in Massachusetts to assess the association between proximity to nuclear facilities and cancer incidence counts. To estimate this relationship, we used separate Generalized Estimating Equation (GEE) Poisson log-linear regression models for each sex and age-group combination (Eq. 1), adjusting for ZIP code-level covariates listed above and detailed in Supplemental Table 3S.

The dependent variable in our models was the annual number of cancer cases, stratified by sex and age group. To account for differences in population size across ZIP codes and demographic subgroups, we included a natural log population offset, ensuring that incidence rates were appropriately scaled by ZIP code, age, sex, and year.

We accounted for the correlation between repeated observations within the same ZIP code over time by specifying ZIP code as the clustering variable and assuming an exchangeable correlation structure, meaning all observations within the same ZIP code share an equal correlation but recognizing that GEEs do not require this correlation structure to be correctly specified in order to obtain unbiased effect estimates. Additionally, we employed robust (sandwich) standard errors to adjust for within-ZIP code correlation and potential overdispersion, ensuring valid statistical inference.

1 $$\begin{aligned}log({E(\lambda }_{ijk}))&={\beta }_{0}+{\beta }_{1}\cdot C1{}_{ij}+{\beta }_{2}\cdot {C2}_{ij}+{\beta }_{3}\cdot {C3}_{ij}\\&+{\beta }_{4}\cdot {C4}_{ij}+\cdots +{\beta }_{p}\cdot {Cp}_{ij}+{\beta }_{e}{E}_{ij}+\\&offset(log(populatio{n}_{ijk}))\end{aligned}$$

where:

- i indexes ZIP codes, j indexes years, and k indexes age-sex groups.
- λ_ijk_ represents the expected number of cancer cases in ZIP code i at year j for age group k.
- C_nij_​ denotes the n^th^ covariate for ZIP code i and year j.
- E_ij_​ represents the sum (over plants) of inverse-distance proximity metric and β_e_​ is the log relative risk characterization the association between the outcome and this metric.
- β_0_​ is the intercept, representing the baseline log cancer incidence rate.

To quantify the burden of cancer incidence attributable to proximity to nuclear power plants, we used the fitted sex- and age-stratified GEE models to calculate the attributable fraction (AF) and attributable cases (AC). AF was computed as (RR—1)/RR, where RR is the estimated relative risk derived as exp (Proximity × Coefficient), using the ZIP code’s proximity value. AC was AF * baseline rate * population. These calculations were performed separately for each unique combination of ZIP code, year, sex, and age group. To estimate AFs for each sex and age group, we summed AC across ZIP codes and years within each stratum and divided by the corresponding total number of observed cases, yielding an average attributable fraction representing the proportion of cancer incidence attributable to proximity within each group.

### Cross sectional model of site-specific cancers

To examine associations between nuclear power plant proximity and site-specific cancer incidence, we conducted a separate cross-sectional analysis. This modeling approach was necessary due to the relatively lower number of cases for individual cancer types after stratification, which limited the stability of annual rate estimates in a spatiotemporal design.

We aggregated the total number of site-specific cancer cases for each ZIP code, stratified by sex and age group, over the full study period (2000–2018). This aggregation removed the temporal component and increased the statistical stability of the models for site specific cancer incidence. Age was collapsed into broader groups (45–65 years and 65 + years) to ensure sufficient counts within each stratum.

For this analysis, we used a log-linear Poisson regression model, applying the same specification described previously (Eq. 1) but excluding the temporal component. Specifically, cancer cases were aggregated across all years (2000–2018) for each ZIP code, stratified by sex and age group. The outcome variable was the total number of site-specific cancer cases for each ZIP code, sex, and age group combination and as in the primary analysis, we included a natural log population (sum of 2000 to 2018 populations for each stratum) offset to account for underlying population size.

The key nuclear power plants proximity variable was the average cumulative proximity to nuclear power plants over the 2000–2018 period, defined as the sum of the inverse distance (1/km) from each ZIP code population center to all active nuclear plants within 120 km for each year, averaged across the period. All covariates were ZIP code-level averages over the same period and included sociodemographic, environmental, and healthcare access factors consistent with the main models (see Supplemental Table 3S).

Separate models were fitted for each cancer site × sex × age group combination.

## Results

### Nuclear power plants proximity

**Fig. 1:**
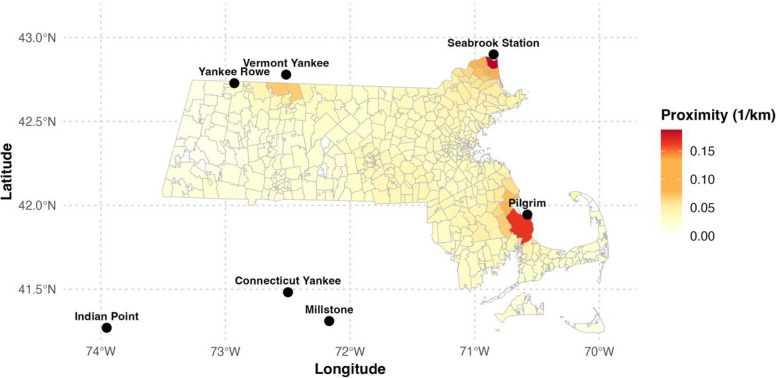
Mean ZIP code level proximity to the seven nuclear power plants within 120 km of Massachusetts (2000–2018)

Figure 1 displays ZIP code level proximity to nuclear power plants used in the cross-sectional model. The figure shows the annual values averaged over the study period to produce a single long-term proximity estimate per ZIP code. The figure also visualizes the locations of all seven nuclear plants within range of the population centers of Massachusetts ZIP codes: Connecticut Yankee, Millstone, Vermont Yankee, Yankee Rowe, Seabrook Station, Indian Point, and Pilgrim.

### All cancers: GEE longitudinal model

**Fig. 2:**
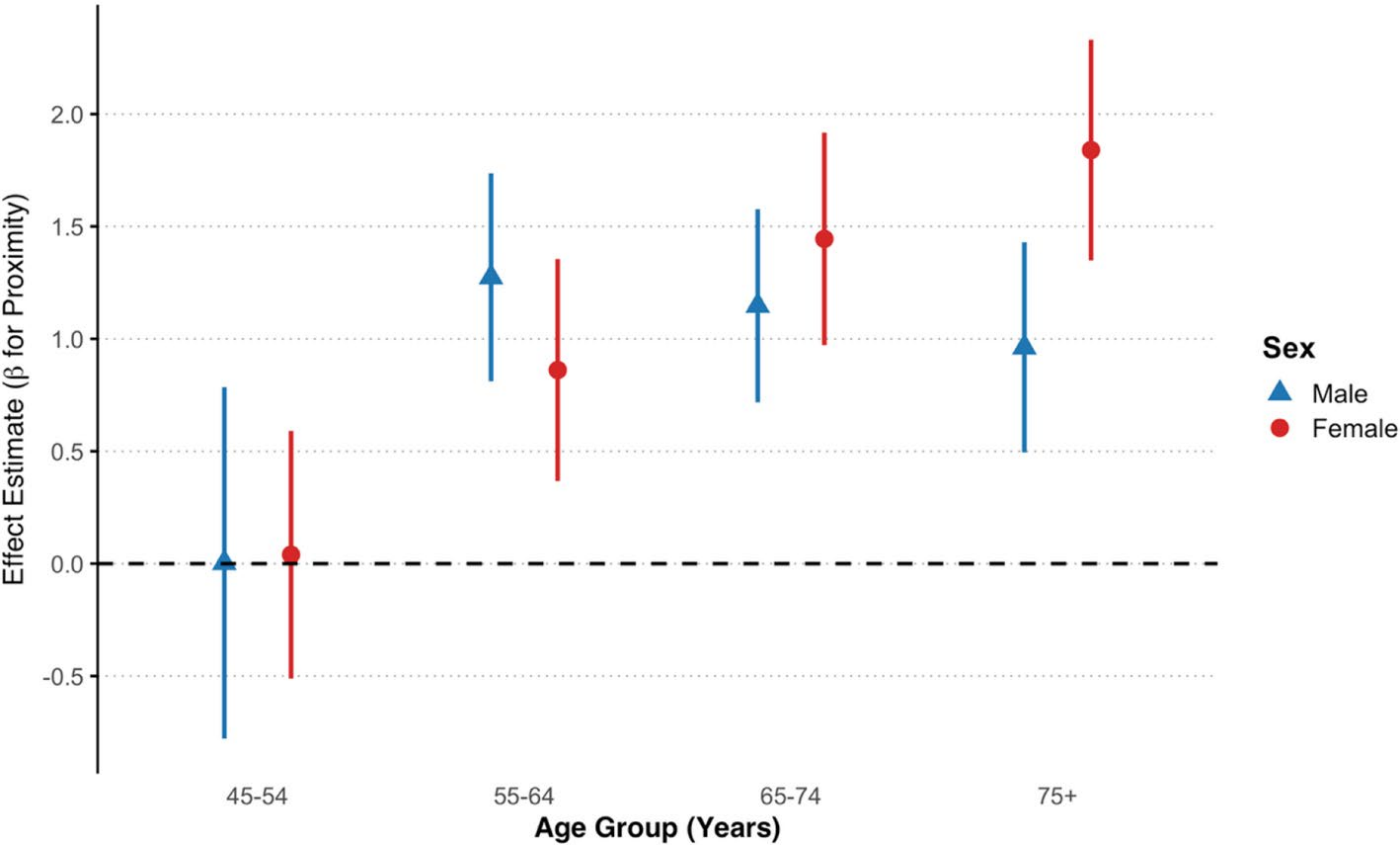
Sex and age group specific associations between nuclear power plants proximity and all cancers incidence in Massachusetts ZIP codes (2000–2018)

Figure 2 presents effect estimates and 95% confidence intervals for the association between nuclear power plant proximity and total cancer incidence across the four age groups, stratified by sex. Among females, a clear positive gradient in effect estimates was observed with increasing age, with the strongest association seen in the 75 + age group. In both sexes, proximity to nuclear power plants was significantly associated with elevated cancer incidence in individuals aged 55 and older. Among males, the largest effect was observed in the 55–64 age group. For the youngest group (45–54), estimates were small and not statistically significant in either sex.

**Fig. 3:**
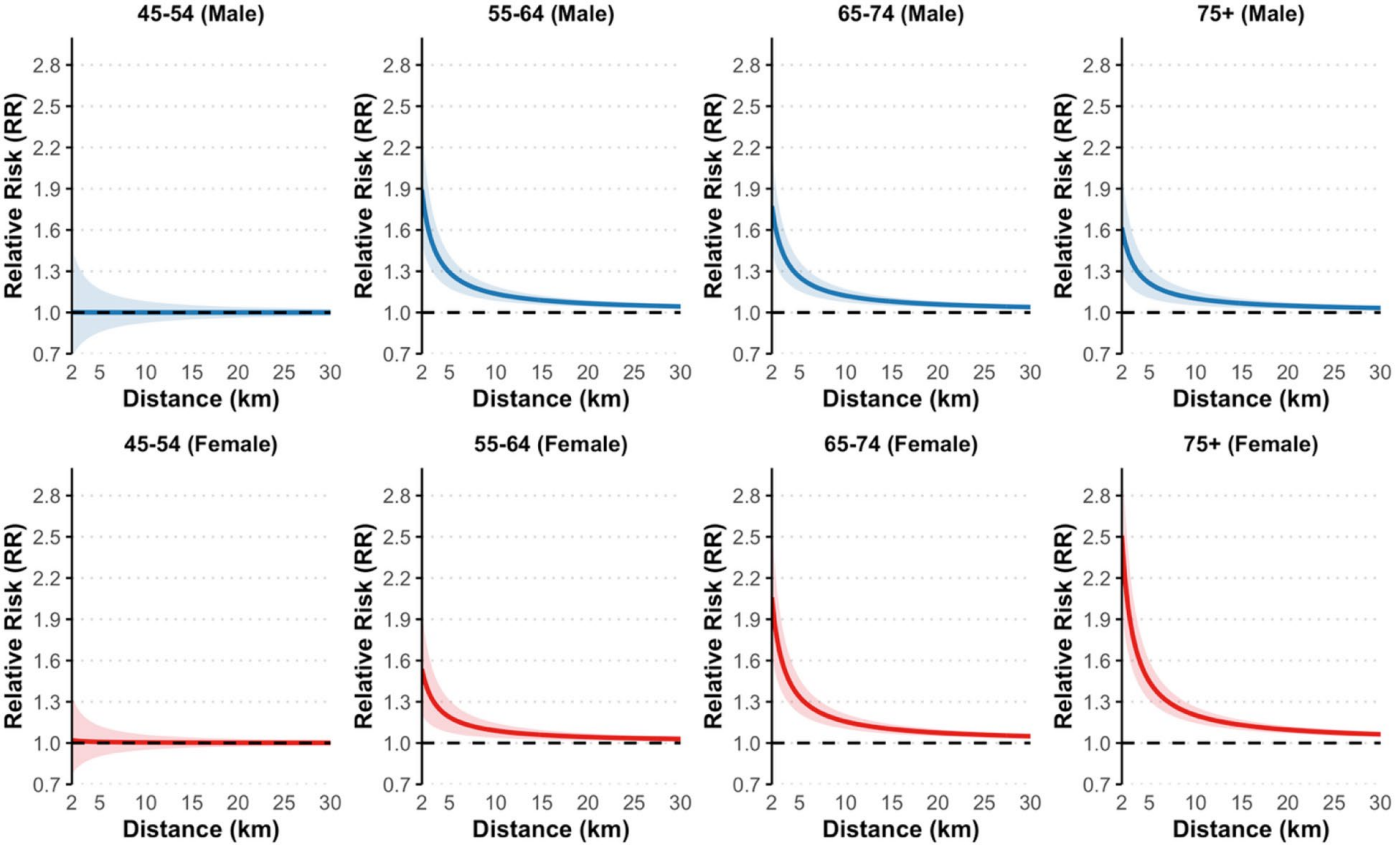
Modeled relative risk of total cancer incidence by distance from a single nuclear power plant, stratified by sex and age group

Figure 3 displays the modeled association between proximity to nuclear power plants and total cancer incidence; shown as relative risk (RR) estimates with 95% confidence intervals across sex and age groups. Each panel corresponds to one of eight sex-age combinations, with distance (from a single plant in km) on the x-axis beginning at 2 km—the approximate minimum observed distance between any ZIP code population center and a nuclear facility. Across all age groups, relative risk generally declined with increasing distance from nuclear plants.

This inverse gradient was more pronounced among females, particularly in the 65–74 and 75 + age groups. At 2 km, the RR was 2.00 (95% CI: 1.59 to 2.52) in females aged 65–74 and 2.53 (95% CI: 1.98 to 3.22) in females aged 75 +. Among females aged 55–64, the RR was also elevated at 1.52 (95% CI: 1.20 to 1.94), while no statistically significant association was found for females aged 45–54 (RR: 1.07, 95% CI: 0.81 to 1.40).

Among males, the strongest associations were observed for those aged 55–64 (RR: 1.97, 95% CI: 1.57 to 2.48) and 65–74 (RR: 1.75, 95% CI: 1.42 to 2.16), with a more modest elevation in the 75 + group (RR: 1.63, 95% CI: 1.29 to 2.06). No statistically significant association was observed for males aged 45–54 (RR: 1.02, 95% CI: 0.70 to 1.50).

The estimated number of cancer cases attributable to nuclear power plant proximity, along with the corresponding attributable fractions (AFs), stratified by sex and age group and derived from the fitted spatiotemporal model, are presented in Table 1. Across all groups, a total of 20,618 cancer cases were attributable to nuclear power plant proximity in Massachusetts from 2000 to 2018.

**Table 1:** Cancer cases attributable to nuclear power plants proximity and their attributable fractions per sex and age group (Massachusetts, 2000–2018)

SEX	AGE GROUP	ATTRIBUTABLE CASES	ATTRIBUTABLE FRACTION
FEMALE	45–54	221 (−705, 1126)	0.5% (−1.5%, 2.4%)
FEMALE	55–64	1,887 (811, 2939)	3% (1.3%, 4.6%)
FEMALE	65–74	3,288 (2215, 4339)	4.8% (3.2%, 6.3%)
FEMALE	75 +	5,419 (4043, 6767)	6.3% (4.7%, 7.8%)
	SUM	10,815 (6364, 15,171)	4.1% (2.4%, 5.7%)
MALE	45–54	49 (−900, 967)	0.1% (−2.7%, 2.9%)
MALE	55–64	3,591 (2395, 4761)	4.8% (3.2%, 6.3%)
MALE	65–74	3,429 (2157, 4678)	3.9% (2.4%, 5.3%)
MALE	75 +	2,734 (1455, 3987)	3.4% (1.8%, 4.9%)
	SUM	9,803 (5107, 14,393)	3.5% (1.8%, 5.2%)

While the average AFs ranged from 2 to 5% across most age–sex strata, substantially higher fractions were observed in communities located closer to nuclear facilities. ZIP codes within 2 to 10 km exhibited markedly elevated relative risks and attributable fractions.

### Site specific cancers: cross sectional model

**Fig. 4:**
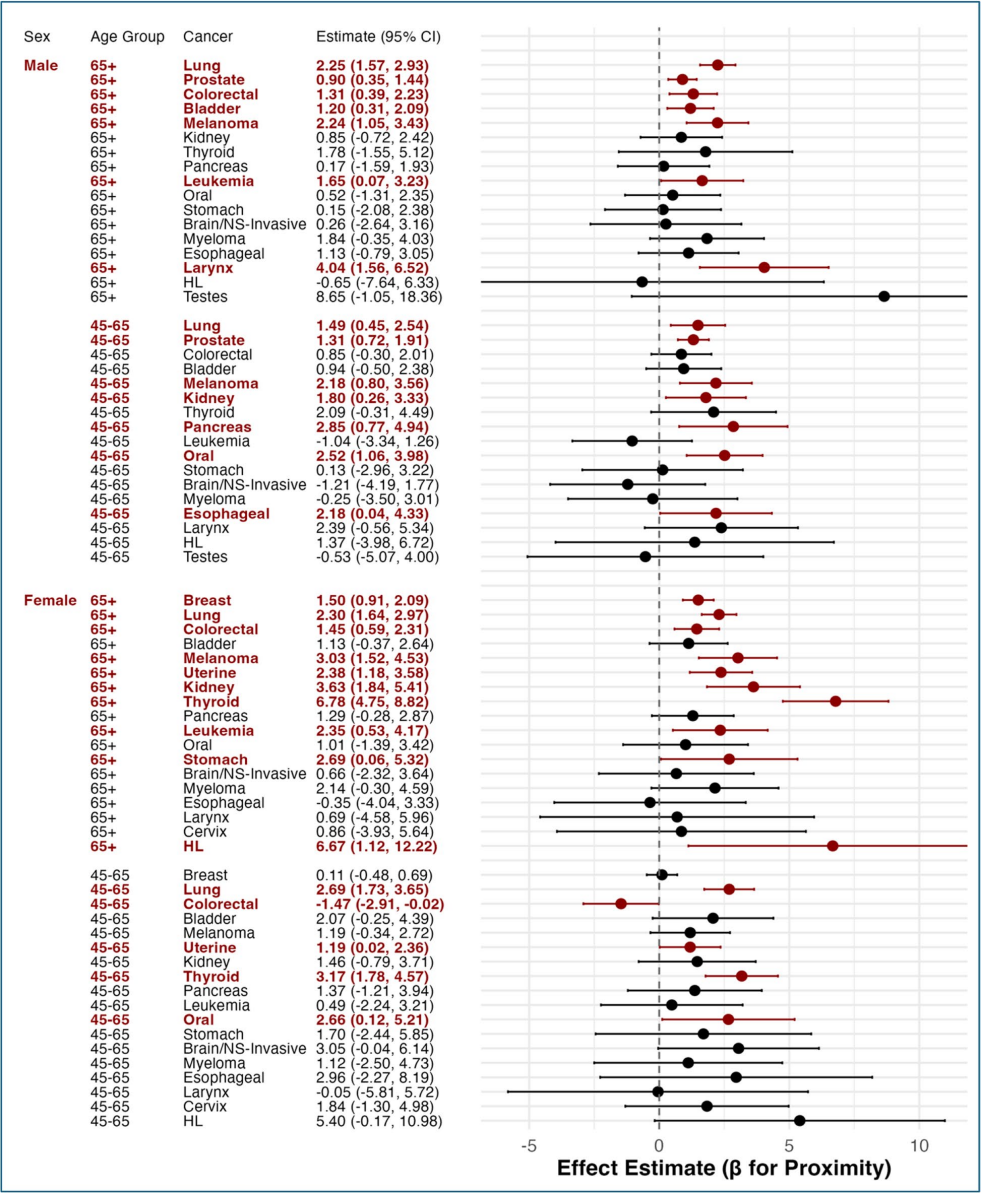
Associations between nuclear power plant proximity and site-specific cancer incidence by sex and age group in the cross-sectional model (significant associations in dark red and not adjusted for multiple comparisons)

Figure 4 displays effect estimates and 95% confidence intervals for the association between ZIP code level proximity to nuclear power plants and site-specific cancer incidence, stratified by sex and age group. The results reveal several strong and statistically significant associations, particularly among older adults.

Among males aged 65 and older, proximity to nuclear power plants was significantly associated with increased incidence of lung cancer (β = 2.25, 95% CI: 1.57 to 2.93), prostate cancer (β = 0.90, 95% CI: 0.35 to 1.44), colorectal cancer (β = 1.31, 95% CI: 0.39 to 2.23), bladder cancer (β = 1.20, 95% CI: 0.31 to 2.09), melanoma (β = 2.24, 95% CI: 1.05 to 3.43), leukemia (β = 1.65, 95% CI: 0.07 to 3.23), and laryngeal cancer (β = 4.04, 95% CI: 1.56 to 6.52).

Among males aged 45 to 65, significant positive associations were observed for lung cancer (β = 1.49, 95% CI: 0.45 to 2.54), prostate cancer (β = 1.31, 95% CI: 0.72 to 1.91), melanoma (β = 2.18, 95% CI: 0.80 to 3.56), kidney cancer (β = 1.80, 95% CI: 0.26 to 3.33), pancreatic cancer (β = 2.85, 95% CI: 0.77 to 4.94), oral cancer (β = 2.52, 95% CI: 1.06 to 3.98), and esophageal cancer (β = 2.18, 95% CI: 0.04 to 4.33).

Among females aged 65 and older, nuclear proximity was significantly associated with increased incidence of breast cancer (β = 1.50, 95% CI: 0.91 to 2.09), lung cancer (β = 2.30, 95% CI: 1.64 to 2.97), colorectal cancer (β = 1.45, 95% CI: 0.59 to 2.31), melanoma (β = 3.03, 95% CI: 1.52 to 4.53), uterine cancer (β = 2.38, 95% CI: 1.18 to 3.58), kidney cancer (β = 3.63, 95% CI: 1.84 to 5.41), thyroid cancer (β = 6.78, 95% CI: 4.75 to 8.82), leukemia (β = 2.35, 95% CI: 0.53 to 4.17), stomach cancer (β = 2.69, 95% CI: 0.06 to 5.32), and Hodgkin lymphoma (HL) (β = 6.67, 95% CI: 1.12 to 12.22).

In the female 45 to 65 age group, significant associations were observed for lung cancer (β = 2.69, 95% CI: 1.73 to 3.65), uterine cancer (β = 1.19, 95% CI: 0.02 to 2.36), thyroid cancer (β = 3.17, 95% CI: 1.78 to 4.57), and oral cancer (β = 2.66, 95% CI: 0.12 to 5.21). In contrast, colorectal cancer showed a borderline negative association (β = –1.47, 95% CI: –2.91 to –0.02).

These results highlight a consistent pattern of elevated cancer risk associated with greater proximity to nuclear power plants, with site-specific and demographic variations.

### Sensitivity analysis

To assess the robustness of our findings, we conducted several sensitivity analyses. First, we re-estimated the primary models using alternative distance thresholds to define the nuclear power plant proximity range. While the main analysis included all nuclear plants within 120 km of ZIP code population centers, we re-ran the models using radii ranging from 80 to 150 km in 10 km increments. The estimated associations were highly consistent across all distance thresholds (Supplementary Fig. 3S), confirming that our results are not sensitive to the specific spatial cutoff used to define exposure.

Second, we evaluated whether the temporal structure of the proximity measure influenced our findings. In the main models, exposure was defined based on the current-year operational status of nearby plants. To account for potential latency effects and cumulative exposure, we repeated the analyses using moving averages of proximity from 1 to 8 years. The estimated associations remained stable across all averaging windows (Supplementary Fig. 1S), indicating that our results are robust to assumptions about latency. As expected, the exposure measures across these windows were highly correlated (Supplementary Fig. 2S), reflecting the limited temporal variability of plant operation status, since exposure changes only when plants come into or go out of operation, and further supporting the temporal stability of our findings.

Finally, we re-ran our models after removing the two ZIP codes with the highest proximity values (02360 and 01952) to ensure that the results were not disproportionately driven by these specific locations. Our findings held up, further supporting the stability of the observed associations.

## Discussion

Our study provides a comprehensive assessment of cancer risks associated with residential proximity to seven nuclear power plants located within 120 km of Massachusetts ZIP codes’ population centers, covering the period from 2000 to 2018. We leveraged both a spatiotemporal model design for all-cancer incidence and a cross-sectional model design for site-specific cancers incidences to evaluate these associations.

In all cancers combined, we observed statistically significant associations between proximity to nuclear power plants and increased incidence of all cancers in older age groups. Our cross-sectional analyses further identified significant associations between nuclear power plant proximity and numerous site-specific cancers, including lung, prostate, colorectal, melanoma, leukemia, thyroid, uterine, and bladder cancers, among others, across different sex and age groups.

Notably, relative risks sharply declined with increasing distance, decreasing substantially at 5 km and becoming negligible beyond approximately 25 km from the nuclear facilities (Fig. 3). This suggests that elevated cancer risks are disproportionately concentrated in communities located within close proximity to nuclear power plants. Unlike health risks associated with coal power plants, which typically affect larger populations spread over broader geographic areas [19], the impacts of nuclear power plants appear to be highly localized, significantly affecting communities residing closest to the plants. Massachusetts, as one of the states with substantial populations residing in close proximity to multiple nuclear power facilities, underscores the importance of these findings.

Previous studies have reported inconsistent associations between proximity to nuclear power plants and cancer risk, likely reflecting differences in plant characteristics, emission controls, and local demographics, as well as substantial methodological variability. Many relied on small samples, limited geographic coverage, or binary proximity metrics that oversimplified exposure contrasts and reduced statistical power. By using a continuous, inverse-distance–weighted exposure metric across all Massachusetts ZIP codes, spanning nearly two decades and leveraging high-quality state cancer registry data, our study provides a more sensitive and spatially refined assessment, helping explain why earlier investigations may have failed to detect associations.

Although previous studies yielded inconsistent conclusions, several international studies have reported significant associations. For instance, in France, proximity to nuclear power plants was associated with increased bladder cancer risk in both sexes [15]. In Germany, elevated risks of leukemia and solid tumors were observed among children residing within 5 km of nuclear facilities [16]. Similarly, studies in South Korea reported significant associations between living within a 5 km radius of nuclear power plants and increased risk of thyroid and breast cancers, as well as other radiation-sensitive cancers, including lung, esophageal, colorectal, kidney, bladder cancers, and leukemia [1, 22]. Research in Spain demonstrated a linear increase in overall cancer risk with decreasing distance to nuclear facilities [28], and an increased risk of developing colorectal and lung cancers among individuals living within 15 km of nuclear fuel facilities [24].

In our national county-level analysis of all-cancer mortality [2], which utilized a similar inverse-distance exposure metric to county population centers across the U.S.,

we found significant positive associations between nuclear power plant proximity and cancer mortality across multiple age groups. Among females, significant associations were observed for all age groups ≥ 45 years, with estimated relative risks at 5 km ranging from 1.09 (95% CI: 1.04 to 1.15) to 1.16 (95% CI: 1.09 to 1.23). Among males, significant associations were found for age groups ≥ 55 years, with relative risks at 5 km ranging from 1.08 (95% CI: 1.03 to 1.14) to 1.15 (95% CI: 1.09 to 1.22). No significant associations were observed among younger males or females aged 35–44.

In comparison, this Massachusetts study on all-cancer incidence identified significant positive associations with nuclear power plant proximity among females aged 55 and older and males aged 55 and older. Relative risks at 5 km among these groups ranged from 1.18 (95% CI: 1.08 to 1.30) to 1.49 (95% CI: 1.32 to 1.60) for females and 1.22 (95% CI: 1.11 to 1.33) to 1.31 (95% CI: 1.20 to 1.44) for males. No significant associations were observed for the youngest age group (45–54) in either sex. These findings align closely with our national mortality study, reinforcing the consistency of associations between nuclear plant proximity and elevated all-cancer risks, despite differences in outcome measures (incidence vs. mortality) and spatial scales (county vs. ZIP code).

Finally, our national county-level mortality analysis of site-specific cancers [3], proximity to nuclear power plants was significantly associated with lung cancer mortality among males aged 45–54, 55–64, 65–74, and 75–84, and among females aged 45–54 and older, including all age groups up to 85 +. For colorectal cancer, significant associations were found among males aged 55–64, 65–74, and 75–84, and females aged 85 and older. For breast cancer, positive associations were observed across all female age groups from 45–54 to 85 +. In comparison, the Massachusetts cancer incidence analysis identified significant associations with lung cancer among males and females aged 45 and older, with colorectal cancer among those aged 65 and older, and with breast cancer among females aged 65 and older. The cancer site-specific analyses (Fig. 4) were included as a complementary component of the study to illustrate the broader trend of the exposure–response relationship across cancer types. These associations were not adjusted for multiple comparisons, and no relative risks were estimated for these specific outcomes.Overall, despite methodological differences in geographic scale (county vs. ZIP code), outcome measure (mortality vs. incidence), and studies design (spatiotemporal vs. cross sectional), these two studies demonstrate substantial concordance, reinforcing concerns about elevated risks of lung, colorectal, and breast cancers associated with proximity to nuclear power plants.

Our attributable fraction calculations further emphasize the considerable public health burden associated with nuclear power plant proximity, with an estimated total of 20,618 attributable cancer cases over the study period. The highest attributable fractions reached approximately 6.3% among older females (75 +), underscoring the pronounced impact on these communities. The strong association observed with lung cancer, the most prevalent and lethal cancer in our analysis [10], suggests inhalation of airborne radionuclides as a potential exposure pathway.

Our study has several limitations. First, our analyses rely on ecological data, which may introduce ecological fallacy, as individual-level exposure variability within ZIP codes is not accounted for. Second, our cumulative inverse-distance proximity measure assumes equal contribution from all nuclear power plants within a 120 km radius and does not incorporate direct radiation measurements (dosimetry), potentially limiting the precision and accuracy of exposure estimation. Third, despite our adjustment for multiple ZIP code-level confounders, residual confounding by unmeasured variables cannot be ruled out. However, our comprehensive adjustment for sociodemographic, environmental, and healthcare access factors helps mitigate this concern. Fourth, we did not incorporate residential histories, potentially introducing exposure misclassification if individuals moved across ZIP codes. Although residential mobility is unlikely to be systematically related to proximity to nuclear plants, such non-differential exposure measurement error would tend to bias our results toward the null. Fifth, our analysis of all cancers combined, despite providing a robust assessment of overall cancer burden, may mask site-specific cancer variations due to different malignancies having varying latency periods and radiation sensitivities. Sixth, we did not examine childhood cancers due to sparse data when stratified by age group, sex, ZIP code, and year. Proper evaluation of childhood cancer risk would require a different analytical approach specifically tailored to rare outcomes. Finally, our analysis could not explicitly account for potential clustering of nuclear power plant workers or nearby residents who may experience higher exposures than those captured by ZIP code-level measures. However, such effects are likely minimal given that plant employees and immediate neighbors represent a very small fraction of the surrounding population, and our sensitivity analysis excluding the closest ZIP codes yielded consistent results.

Nevertheless, our study has important strengths. By employing both a GEE longitudinal model for overall cancer incidence and cross-sectional models for specific cancers, we provided robust evidence for associations between nuclear power plant proximity and cancer risks. Our analysis leverages Massachusetts’ high-quality, comprehensive cancer registry data, allowing precise estimation of cancer incidence at fine spatial scales. Furthermore, our inverse-distance exposure metric represents a methodological advancement over traditional fixed distance cutoff models, reducing potential exposure misclassification and strengthening the validity of observed associations. Additionally, detailed covariate adjustments for demographic, socioeconomic, environmental, and healthcare access factors enhance the robustness of our findings by minimizing confounding bias.

Given the renewed policy-driven interest in expanding nuclear power in the United States, our findings underscore the need for continued research and enhanced public health surveillance near nuclear facilities. Future studies should refine exposure assessment using direct radiation monitoring, dispersion modeling, and residential history data, and employ longitudinal designs to better evaluate latency and site-specific cancer risks. Strengthening emission controls, improving environmental monitoring, and prioritizing research and surveillance within approximately 25–30 km of nuclear plants will be essential for advancing evidence-based protection of nearby communities.

## Conclusion

We observed increased cancer incidence among Massachusetts residents living closer to nuclear power plants, with the strongest associations found in older adults. Cancer site-specific analyses identified elevated risks for numerous cancers, including lung, prostate, breast, colorectal, bladder, melanoma, leukemia, thyroid, uterine, kidney, laryngeal, and pancreatic cancers, among others, with variations by sex and age group. These results closely align with our previous national-level studies, reinforcing concerns about the health impacts of residential proximity to nuclear facilities. With the renewed interest in expanding nuclear power for decarbonization and energy security, our findings underscore the importance of integrating public health considerations into nuclear energy policies, strengthening surveillance efforts, and conducting further research to elucidate specific exposure pathways and strengthen the basis for causal interpretation of these results.

## Supplementary Information

Supplementary Material 1.

## Data Availability

The cancer incidence data utilized in this study was obtained from the Massachusetts Cancer Registry (MCR) and is confidential; therefore, it cannot be shared by us. Interested parties can apply to the MCR for access. All other data sources are publicly available. Sample code for the statistical epidemiological models used in our analysis can be provided upon request.
