# Supplementary material for: Residential proximity to nuclear power plants and cancer incidence in Massachusetts, USA (2000–2018)
Source: Environ Health. 2025 Dec 18;24:92. doi: 10.1186/s12940-025-01248-6 (PMC12713251; doi:10.1186/s12940-025-01248-6)
Supplement: Supplementary file 1 — Supplementary Material 1. [file 12940_2025_1248_MOESM1_ESM.docx]

**Supplementals**

**Table 1S:** Cancer cases count for the study duration (2001-2018) and cases per year in Massachusetts.

| Cancer Type | Total Cases | Cases per Year (SD) |
| --- | --- | --- |
| Breast | 106,968 | 5629.9 (539) |
| Lung | 97,582 | 5135.9 (257) |
| Prostate | 95,057 | 5003 (551) |
| Colorectal | 67,199 | 3536.8 (520) |
| Bladder | 36,158 | 1903.1 (74) |
| Melanoma | 31,289 | 1646.8 (239) |
| Uterine | 23,472 | 1235.4 (169) |
| Thyroid | 22,866 | 1203.5 (331) |
| Kidney | 22,590 | 1188.9 (190) |
| Leukemia | 18,927 | 996.2 (155) |
| Pancreas | 18,831 | 991.1 (180) |
| Oral | 17,319 | 911.5 (157) |
| Stomach | 10,334 | 543.9 (37) |
| Brain/Ns-Invasive | 10,077 | 530.4 (77) |
| Myeloma | 9,329 | 491 (113) |
| Esophageal | 8,816 | 464 (43) |
| Larynx | 5,240 | 275.8 (23) |
| Hl | 4,115 | 216.6 (19) |
| Cervix | 4,038 | 212.5 (21) |
| Testes | 3,874 | 203.9 (17) |

**Table 2S:** Total and Annual Cancer Case Counts by Age Group and Sex (Massachusetts, 2000–2018).

| sex | age group | total cases | mean yearly cases | sd yearly cases |
| --- | --- | --- | --- | --- |
| Female | 0-1 | 83 | 4.4 | 2.7 |
| Female | 1-4 | 474 | 24.9 | 5.2 |
| Female | 5-14 | 2289 | 120.5 | 20.1 |
| Female | 15-24 | 7639 | 402.1 | 50.5 |
| Female | 25-34 | 21541 | 1133.7 | 55.5 |
| Female | 35-44 | 48631 | 2559.5 | 173.6 |
| Female | 45-54 | 954 | 50.2 | 10.1 |
| Female | 55-64 | 66341 | 3491.6 | 521.2 |
| Female | 65-74 | 71358 | 3755.7 | 628.6 |
| Female | 75+ | 88310 | 4647.9 | 187 |
| Male | 0-1 | 96 | 5.1 | 2.6 |
| Male | 1-4 | 550 | 28.9 | 8.1 |
| Male | 5-14 | 2034 | 107.1 | 15.3 |
| Male | 15-24 | 4440 | 233.7 | 29.6 |
| Male | 25-34 | 9543 | 502.3 | 47.1 |
| Male | 35-44 | 34903 | 1837 | 132 |
| Male | 45-54 | 859 | 45.2 | 8.3 |
| Male | 55-64 | 78480 | 4130.5 | 400.3 |
| Male | 65-74 | 91901 | 4836.9 | 541 |
| Male | 75+ | 83609 | 4400.5 | 307.5 |

**Table 3S**. Annual covariates used in the statistical models (2000-2018).

| Variable | Data source |
| --- | --- |
| Education below high school, % | United States Census American Community Survey |
| Median household income, $ | United States Census American Community Survey |
| Below federal poverty level, % | United States Census American Community Survey |
| White, % | United States Census American Community Survey |
| Population density, ppl/km^2^ | NASA Socioeconomic Data and Applications Center |
| Average temperature, degrees ^o^C | National Oceanic and Atmospheric Administration |
| Average relative humidity, % | National Oceanic and Atmospheric Administration |
| Current smoke, (1-100) | BRFSS Area Health Resource Files |
| Nearest hospital, Km | ESRI’s hospital distribution files |
| Age over 65, % | United States Census American Community Survey |
| Renting, % | United States Census American Community Survey |
| Poverty, % | United States Census American Community Survey |
| Asian, % | United States Census American Community Survey |
| African American, % | United States Census American Community Survey |
| Average BMI | BRFSS Area Health Resource Files |
| PM2.5 | Atmospheric Composition Analysis Group – Washington University in St. Louis |

**Table 4S:** All power plants within 120 km of a ZIP code population center in Massachusetts that were operational for at least one year in the period 2000 to 2018.

| Plant Name | Reactor Type | Commercial Operation | Closed | State |
| --- | --- | --- | --- | --- |
| Connecticut Yankee | PWR | 1/1/68 | 12/5/96 | Connecticut |
| Millstone | BWR, PWR | 12/28/70 | NA | Connecticut |
| Vermont Yankee | BWR | 11/30/72 | 12/29/14 | Vermont |
| Yankee Rowe | PWR | 7/1/61 | 10/1/91 | Massachusetts |
| Seabrook Station | PWR | 3/15/90 | NA | New Hampshire |
| Indian Point | PWR | 10/1/62 | 4/30/20 | New York |
| Pilgrim | BWR | 12/9/72 | 5/31/19 | Massachusetts |


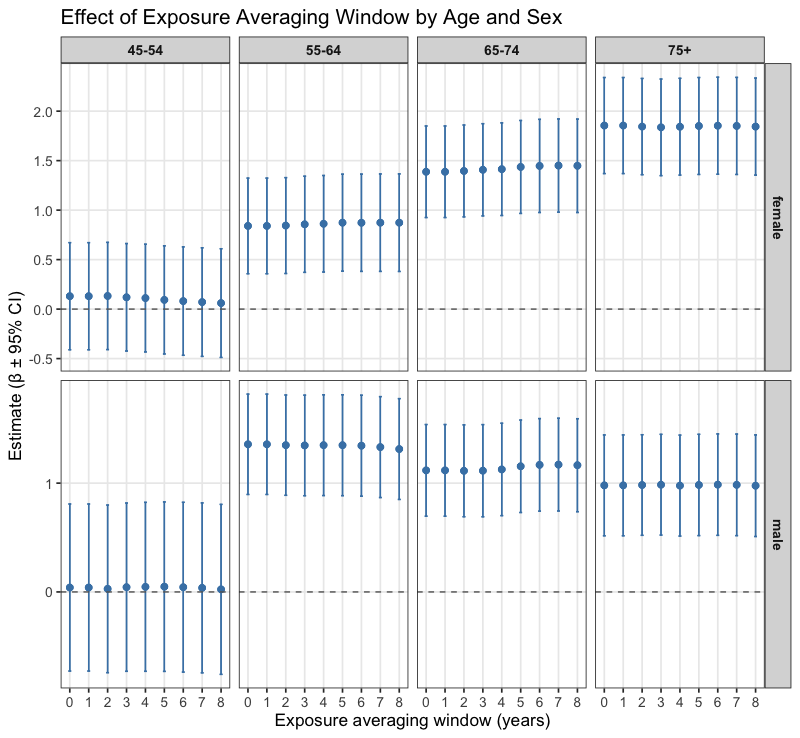


Figure 1S: Comparison of estimated associations across exposure averaging windows (0–8 years) by age group and sex.


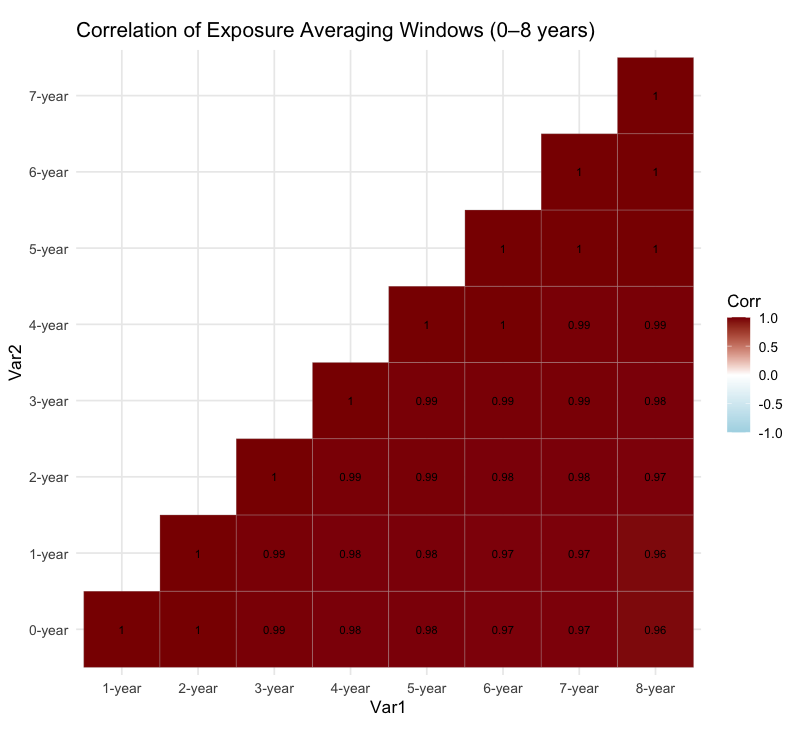


Figure 2S: Correlation matrix of exposure measures across averaging windows (0–8 years).


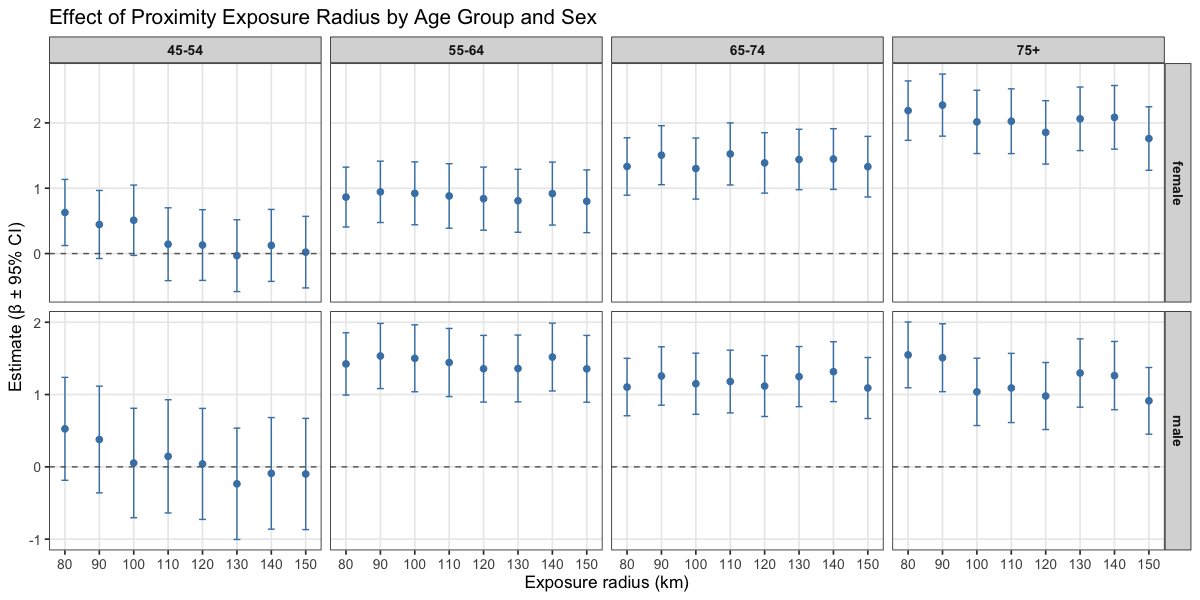


Figure 3S: Sensitivity analysis of exposure radius in relation to cancer incidence by age group and sex.
